# Social and health outcomes following upgrades to a national housing standard: a multilevel analysis of a five-wave repeated cross-sectional survey

**DOI:** 10.1186/s12889-017-4928-x

**Published:** 2017-12-02

**Authors:** Wouter Poortinga, Nikki Jones, Simon Lannon, Huw Jenkins

**Affiliations:** 1 Welsh School of Architecture, Cardiff University, Bute Building, King Edward VII Avenue, Cardiff, Wales CF10 3NB UK; 2 School of Psychology, Cardiff University, Tower Building, 70 Park Place, Cardiff, Wales CF10 3AT UK

**Keywords:** Housing standards, Housing quality, Fuel poverty, Respiratory health, Mental health

## Abstract

**Background:**

While existing research indicates that housing improvements are associated with health improvements, less is known about the wider social and health benefits of meeting national housing standards, as well as those of their specific constituent measures. This study evaluates the impacts of a managed housing upgrade programme through a repeated cross-sectional survey design.

**Methods:**

A five-wave repeated cross-sectional survey was conducted over a seven-year period from 2009 to 2016 (*n* = 2075; *n* = 2219; *n* = 2015; *n* = 1991; and *n* = 1709, respectively). The study followed a managed upgrade programme designed to meet a national social housing standard over an extended period. The data were analysed from a multilevel perspective to take account of the time-dependent nature of the observations and differences in socio-demographic composition.

**Results:**

The installation of the majority of individual housing measures (new windows and doors; boilers; kitchens; bathrooms; electrics; loft insulation; and cavity/external wall insulation) were associated with improvements in several social (housing suitability, satisfaction, and quality; thermal comfort and household finances) and health (mental, respiratory and general health) outcomes; and analyses showed relationships between the number of measures installed and the total amount invested on the one hand and the social and health outcomes on the other. There were however a few exceptions. Most notably, the installation of cavity wall insulation was associated with poorer health outcomes, and did not lead to better social outcomes. Also, no association was found between the number of measures installed and respiratory health.

**Conclusions:**

The study suggests that substantial housing investments through a managed upgrade programme may result in better social and health outcomes, and that the size of the improvements are proportionate to the number of measures installed and amount invested. However, there may be risks associated with specific measures; and more attention is needed for mechanical ventilation when upgrading energy efficiency of houses through fabric work. In addition to providing new evidence regarding the wider social and health outcomes, the study provides an analytical approach to evaluate upgrade programmes that are delivered over multiple years.

## Background

Poor quality housing may contribute to social and health inequality that could be addressed through housing improvements [1–3]. The current evidence suggests that housing improvements can lead to a number of positive health outcomes, in particular when they are targeted at those with existing poor health and inadequate warmth [3–5]. However, the benefits of area-led housing interventions are still uncertain [3, 6], and much remains to be learnt about the impacts of meeting housing quality standards and their constituent measures [4, 7]. Studying the impacts of wider upgrade programmes to meet standards is more challenging than those of targeted affordable warmth interventions, as upgrade programmes often take place over a longer period (i.e. years rather than weeks or months). Changes in health may then reflect changes in the composition of the population rather than result from the housing upgrades themselves. Housing upgrade programmes also often involve multiple measures beyond the regular affordable warmth ones. A recent systematic review concluded that most studies examine the overall impacts of housing improvement programmes, and that less is known about the health effects of specific measures and/or extent of the intervention [3]. Thus far, only a limited number of studies have tried to disentangle the impacts of programmes consisting of multiple measures [4, 8]. Furthermore, research has largely ignored the social benefits that may be part of the pathways to health [9, 10]. Evidence from both qualitative and quantitative research suggests that housing upgrade programmes can result in a wide range of positive outcomes related to housing conditions [11], fuel poverty [9], thermal comfort [11, 12], financial stress [13–15], and subjective wellbeing [11].

This paper addresses some of the identified gaps in the literature. It reports on a seven-year evaluation study that aimed to examine the wider social and health benefits of a managed housing upgrade programme to meet a national housing standard over an extended period of time. The study’s objectives were to examine (1) the changes in a range of social and health outcomes following upgrades to a national social housing standard, and (2) whether these outcomes can be linked to the specific measures that were part of the upgrade programme. The social and health outcomes are in the areas of (a) housing suitability, satisfaction, and quality, (b) thermal comfort and household finances, and (c) mental, respiratory and general health. In addition to providing new evidence regarding the wider social and health benefits of housing upgrades, the paper presents an analytical approach to examine such outcomes through a repeated cross-sectional survey design. The approach allows the different outcomes to be linked to the individual measures and the extent of the intervention. The context and details of the upgrade programme are described in the Methods section; and below we discuss the expected social and health outcomes of the programme.

### Pathways from housing upgrades to social and health outcomes

The literature has identified several pathways that may link housing upgrades to social and health outcomes [8–11, 16]. The *two main pathways* associated with energy efficiency improvement involve better *thermal living conditions* and improved *heating affordability* [9–11]. Past research has found associations between living in cold and damp housing and mental wellbeing, whereby such homes potentially contribute to a variety of mental health stressors [2, 16–18]. Stress may arise from thermal discomfort, concerns about household finances and debt, and even from worries about the health effect of living in a cold and damp home itself [13]. There is now extensive empirical evidence that energy performance investments can produce better mental wellbeing, in particular when they are targeted at vulnerable populations [3, 16], via the pathways of thermal satisfaction and improved household finances [11]. Evidence of improvements in general health following warmth interventions has also been found, although this is not always statistically significant [3]. It is however anticipated that broad improvements in living conditions and mental wellbeing are likely to be associated with better general health [10, 16, 19].

In addition to posing a risk to mental health, cold and damp housing may also contribute to poor respiratory health [2, 20–22]. Poor internal conditions can affect respiratory health in several ways. Low temperatures may suppress the function of the immune system [23], raising the risk of respiratory problems [17]. Low indoor temperatures may also encourage the development of damp and mould in the home [24]. Inadequate heating, insulation and ventilation can therefore lead to exposure to allergenic spores, a known risk factor for asthma sufferers [25]. A combination of measures can improve internal conditions [12], and as a result lower the allergy burden [26]. It is therefore likely that the warmth improvements that are part of the upgrade programme will lead to fewer housing-related problems, such as mould and damp, as well as improved respiratory health. More recently, there have been indications that energy efficiency improvements may produce adverse health outcomes, in particular where houses are built or upgraded to high standards of insulation and air tightness [27]. In Wales, which has a wet climate, this may lead to higher indoor humidity levels and as a result mould contamination. Highly energy efficient homes have been linked to increased asthma in adults [22]. This may however not be due to internal hydrological conditions, as the same study found that higher energy efficient homes had lower levels of mould growth [22]. Other research suggests that energy-efficiency retrofits can improve occupants’ health through reduced exposure to cold and pollutants, but that their benefits will be reduced if not accompanied by appropriate ventilation [28]. A recent study illustrated the importance of mechanical ventilation when making buildings more airtight [12]. Increases in indoor relative humidity levels were only found for buildings that received cavity-wall insulation (which generally was installed without mechanical ventilation), not in British steel-framed buildings or in buildings with solid walls receiving external wall insulation (which was accompanied by extractor fans).

There is now a substantial literature on the health outcomes following affordable warmth interventions. However, research on other housing improvements is less well developed. Only a handful of (mostly qualitative) studies examined outcomes associated with internal improvements, such as providing up-to-date kitchens and bathrooms [14, 15, 29, 30]. Caldwell and colleagues reported increased use of the kitchen following upgrades, as well as increased thermal comfort [3]. Gilbertson and colleagues similarly reported increased use of the kitchen and improved nutrition [15], which may result in better general health outcomes in the longer term. Research conducted as part of the GoWell programme found that the provision of new kitchens and bathrooms may produce better mental health outcomes [4], although another study using data linkage found no change in emergency admissions following kitchens or bathroom upgrades [7]. Evidence relating to other improvements, such as rewiring, is even more scarce, not least because they are often installed alongside other internal improvements. As noted by Thomson and colleagues, housing-led renewal programmes are often analysed as a whole, and not by specific measures and/or the extent of intervention [3]. While rewiring may reduce the number of accidents within the home, it is less likely to have strong associations with mental, respiratory and general health outcomes [7]. Given the paucity of good quality evidence, the suggested pathways to health from these and other housing improvements can only be speculative at this stage. Conceptual frameworks used in other research posits that updating kitchens and bathrooms may produce better mental and general health outcomes through better designed facilities, and better internal appearances leading to improved housing satisfaction [7, 8, 10].

### Expected associations of housing improvements

Table 1 presents the hypothesised associations of the housing improvements that will be tested in this research. The warmth measures that are part of the upgrade programme include wall and loft insulation, boilers, and (double-glazed) windows and doors. In line with previous research, installation of these measures is expected to be associated with better mental, respiratory and general health via the two main pathways of thermal satisfaction and improved household finances. In addition, all measures that improve the energy performance of the buildings are expected to be associated with fewer housing-related problems, such as mould and damp. While not all types of insulation were accompanied by mechanical ventilation (external wall insulation was installed with and cavity wall insulation without mechanical ventilation), it is expected that they all lead to fewer building-related problems due to improved internal hydrothermal conditions; the standard of a SAP 65^1^ energy rating is not expected to bring about problematic levels of air tightness. While new kitchens, bathrooms and electrics, are expected to be associated with better mental and general health, due to tenants being more content and at ease in their homes, there is no empirical evidence or theoretical reasons to believe that these measures eliminate damp and mould, and as a result will help to improve respiratory health. The measures are equally not expected to produce large-sized improvements in thermal comfort and household finances.

**Table 1:** Hypothesised associations of the intervention measures and overall upgrade programme with the different social and health outcomes

	Housing suitability and satisfaction	Housing quality	Thermal comfort	Household finances	Mental health	Respiratory health	General health
Windows and doors	✓	✓	✓	✓	✓	✓	✓
Boilers	✓	✓	✓	✓	✓	✓	✓
Kitchens	✓	–	–	–	✓	–	✓
Bathrooms	✓	–	–	–	✓	–	✓
Electrics	✓	–	–	–	✓	–	✓
Loft and wall insulation	✓	✓	✓	✓	✓	✓	✓
Overall programme	✓	✓	✓	✓	✓	✓	✓

All measures of the upgrade programme are expected to be linked to housing satisfaction. That is, any investment that improves the quality or appearance of the house will be appreciated by the tenants, and are therefore likely to lead to higher suitability and satisfaction ratings. This not only applies to the energy efficiency measures, such as wall insulation and new boilers, but also to windows and doors with improved safety features, up-to-date kitchens and bathrooms with appropriate design, layout and state of repair, and electrics that involve the installation of power sockets, Carbon Monoxide and Smoke detectors, and security lighting. All of these features are likely to make tenants feel more content, safe, and at ease in their homes.

The overall intervention (as indicated by the number of measures and total amount invested; see below) is expected to be positively associated with all outcome measures.

## Methods

### Study context

#### Location

The study was conducted in Carmarthenshire, Wales, one of the largest Welsh unitary authorities located in the south-west of Wales. Carmarthenshire is a largely rural Authority, with three significant urban centres (Llanelli, Carmarthen, and Ammanford). There is significant deprivation within Carmarthenshire, with 25 LSOAs (lower layer super output areas)^2^ within the 30% most deprived areas in Wales. The majority of these areas are located within urban areas [31].

#### The housing upgrade programme

The Carmarthenshire Homes Standard (“the upgrade programme”) was developed following the publication of the national housing strategy for Wales, which requires all social housing landlords, including local authorities, to bring all social housing up to the Welsh Housing Quality Standard [32, 33]. The standard sets out a basic set of requirements to ensure that all social housing in Wales is in a good state of repair; safe and secure; adequately heated; fuel efficient and well insulated; well managed; and located in attractive and safe environments. While similar standards have been introduced in the other UK countries, the detailed requirements of the Welsh Housing Quality Housing Standard means that it is more challenging to meet than those set elsewhere [7].

Following a consultation with their tenants in 2005, Carmarthenshire County Council (“the council”) took the decision to retain ownership of their social housing stock and to set its own, slightly higher housing standard. The standard was to be achieved through a managed upgrade programme running from 2007 to 2015.

Properties received a number of improvements depending upon the state of repair relative to the housing standard. These improvements were delivered in a work programme with extensive tenant consultation and engagement. It was agreed that the programme was to be spread evenly across the authority on an *area-by-area* basis, and conducted in such a way that work could be carried out with the tenants in situ, in order to minimise the disruption to them. Tenants were informed at the start of the programme when the work would take place.

The programme involved the elements of (1) windows and doors^3^; (2) boilers; (3) kitchens^3^; (4) bathrooms^4^; (5) electrics^5^; (6) loft insulation^6^; (7) cavity-wall insulation^6^; (8) external wall insulation^6^; and (9) safety improvements to external paths. The council also developed an environmental work package to identify and improve elements in the in their wider estate environment. This part of the programme was however scaled down at an early stage due to budget constraints.

The elements were delivered through four broad work packages, namely (1) *external work* (windows and doors); (2) *internal work* (kitchens, bathrooms, boilers, electrics); (3) *thermal insulation/external finishes*; and (4) *gardens and estates*. This means that multiple measures were installed as part of the same work package, although not all houses received all of the elements mentioned. More than 9000 properties were upgraded at an overall cost of £200+ million. Virtually all properties were compliant by the beginning of 2016, at the time of the fifth and final survey of this study.

### Research design

#### The health surveys

A study was designed to assess the social and health benefits to tenants resulting from the housing improvements. In the absence of appropriate comparator groups within the area, it was decided to follow the tenants throughout the upgrade programme with a series of health surveys. The design consisted of *five repeated cross-sectional health surveys* conducted over a seven-year period from 2009 to 2016. The sampling frame of the survey were social housing tenants of the council. The social housing stock of the council consisted of approximately 9200 properties at the time of the study, of which about 400 houses were of non-traditional build (e.g. steel-framed or concrete constructions). All addresses were approached for either a postal survey or face-to-face interviews. Any adult tenant currently living in the council-owned properties was eligible for inclusion. Responses from non-traditional properties were excluded from the analyses, to ensure that the results of the study are generalisable to other programmes upgrading standard social housing in the UK.

The first wave of data collection took place in early 2009 as part of a pilot study. Following the successful delivery of the pilot study, four additional health surveys were conducted in 2011, 2012, 2014 and 2016.^7^ All five surveys that are part of the study were conducted in the same period within the heating season. The fifth and final survey was conducted at the end of the programme, when most of the improvements were completed. Data were collected using postal questionnaire and face-to-face interviews by two Wales-based market research companies with established networks of trained interviewers. The project received ethical approval from the School Research Ethics Committee of the Welsh School of Architecture.

In the first survey, conducted in 2009, tenants of properties where work had not started yet were contacted for face-to-face interviews. All other tenants were approached by postal questionnaire. All tenants who took part in the first survey were contacted to be interviewed face-to-face for the second survey in 2011. The remaining tenants were contacted by postal questionnaire. Respondents who had not consented to be re-contacted were excluded from the study. This sampling strategy was repeated in the remaining surveys to ensure that the samples were comparable across the different years. The response rates were generally higher for the face-to-face interviews than for the postal questionnaires.^8^ In all five surveys, a prize draw was offered as an incentive for residents to complete and return their postal questionnaires. The number of households contacted decreased over the seven-year period of the study. This was mostly due to the exclusion of tenants who did not want to be contacted again.

The survey was designed to cover the topics of (1) housing suitability, satisfaction, and quality, (2) thermal comfort and household finances, and (3) mental, respiratory and general health. The three topics are used as a structure for this paper.

Data from the five health surveys were combined to form a single dataset, and subsequently linked to intervention data held by the council (see *The intervention dataset* section). The data were linked using a unique property reference number, which was included in the surveys and in the intervention dataset. The reference number enabled the properties to be matched with deprivation data from the Welsh Index of Multiple Deprivation. After removal of multiple responses from the same household and of responses from tenants living in non-traditional housing, the overall dataset consisted of 10,009 individual responses. Respondent characteristics of the five surveys are provided in Table 2.

**Table 2:** Socio-demographic characteristics of the respondents to the five surveys (%)

	2009 (n = 2075)	2011 (*n* = 2219)	2012 (*n* = 2015)	2014 (*n* = 1991)	2016 (*n* = 1709)
Male	31	35	36	35	35
Age under 36	15	15	15	15	12
Age 36–45	12	11	10	10	10
Age 46–54	11	11	12	11	12
Age 55–64	17	16	17	16	16
Age 65+	41	44	45	48	48
No qualification	39	56	58	54	49
Housing benefit recipient	61	68	70	70	68
WIMD quartile 1	19	24	20	21	18
WIMD quartile 2	37	36	36	37	38
WIMD quartile 3	32	29	31	30	31
WIMD quartile 4	13	12	13	13	14
Retired	35	43	45	47	43
Not working other	36	36	37	33	34
Current smoker	34	33	34	30	29
Past smoker	29	29	24	29	29
Face to face interview	15	44	50	52	53
Postal questionnaire	85	56	50	48	47

#### The intervention dataset

Records on progress of the upgrade programme were used to determine changes in the status of *eight housing intervention measures*, i.e. (1) new windows and doors, (2) boilers, (3) kitchens, (4) bathrooms, (5) electrics, (6) loft insulation, (7) cavity-wall insulation, and (8) external wall insulation. Measures that were part of the gardens and estates work package were not included because this part of the programme was scaled down at an early stage. For each survey, properties were categorised according to whether an intervention had taken place or not, for each of the listed measures. Table 3 shows the number and percentage of completed measures in the five surveys.

**Table 3:** Number and percentage of completed individual housing intervention measures in the five surveys

Measure	2009 (n = 2075)	2011 (n = 2219)	2012 (n = 2015)	2014 (n = 1991)	2016 (n = 1709)
Windows and doors	1083 (52%)	2138 (96%)	1996 (99%)	1983 (100%)	1709 (100%)
Boilers	375 (18%)	878 (40%)	985 (49%)	1455 (73%)	1435 (84%)
Kitchens	248 (12%)	762 (34%)	864 (43%)	1466 (74%)	1551 (91%)
Bathrooms	327 (16%)	872 (39%)	945 (47%)	1543 (78%)	1581 (93%)
Electrics	318 (15%)	903 (41%)	968 (48%)	1549 (78%)	1628 (96%)
Loft insulation	1129 (55%)	1754 (79%)	1611 (80%)	1608 (81%)	1381 (81%)
Cavity wall insulation	1533 (74%)	1706 (77%)	1554 (77%)	1581 (79%)	1385 (81%)
External wall insulation	110 (5%)	154 (7%)	184 (9%)	200 (10%)	222 (13%)
Number of measures per property (SD)	2.46 (1.51)	4.13 (1.83)	4.52 (1.88)	5.72 (1.63)	6.37 (1.02)
Total spend: £k/property (SD)	5.45 (5.29)	10.56 (6.70)	12.09 (6.91)	16.28 (5.95)	18.67 (4.37)

The number of improvements installed in each property varied according to the condition of the property at the start of the study. Some properties were already compliant for some or all of the measures at the time of the first survey in 2009. Where properties had received measures prior to the first survey, they were categorised as having received the intervention. The timing of the work programme was not related to residents’ needs in terms of health, housing condition, or other personal characteristics, and can therefore be assumed effectively random. Where properties had received measures prior to the first survey round, they were still categorised as receiving an intervention and the date was recorded for the installation of each measure.

The intervention data were further used to calculate a *number of measures* variable. This variable indicates how many different intervention measures (out of the eight housing measures of interest) had been installed within each property at the time the five surveys were conducted. The maximum number of possible measures in any one property was seven (a property could only receive external wall *or* cavity-wall insulation).

A *total spend* variable was created reflecting the average total investment of the programme. The variable was calculated for each individual property at the time of the five surveys and expressed in thousand pounds per property (£k/property), using cost data provided by the council. The *total spend* variable is closely related to the *number of measures* variables, but weighted according to the cost of the individual measures.^9^ This is based on the assumptions that the cost of the measure is indicative of its size and therefore potential impact on the outcome measures.

#### Outcome measures

The study focused on *nine outcome measures* relating to the topics of housing suitability, satisfaction, and quality; thermal comfort and household finances; and mental, respiratory and general health.

Three measures were used to indicate housing suitability, satisfaction, and quality, respectively.

*Suitability of housing* was measured by asking the tenants “Do you feel that your home is suitable for the needs of you and your family?” Response options were “yes” (1) or “no” (0).

*Satisfaction with the state of repair* of the home was measured by asking “In general, how satisfied or dissatisfied are you with the current state of repair of your home?” The 5-point answering scale ranged from “very dissatisfied” (1) to “very satisfied” (5), with “neither satisfied nor dissatisfied” as the scale midpoint (3).

A *building problems* variable was constructed as an indicator of *housing quality*. Respondents were asked whether they currently experience any of the following problems in their home: draught, condensation, damp on walls and/or floors, mould, lack of adequate heating, rot in windows or doorframes, and leaking roof. This variable was dichotomised to indicate whether someone had experienced *any building problems* (1) or not (0).

Three measures were used to indicate thermal comfort and household finances.

*Thermal comfort* was measured by asking tenants the extent to which they were satisfied or dissatisfied with the temperature in (1) the main living room during the day, (2) the main living room in the evening, and (3) the bedroom at night on a typical winter’s day. Scale analyses showed that the reliability of the scale is consistently high across the five surveys (Cronbach’s α > 0.90). The three items were therefore combined into a single thermal comfort scale ranging from 1 (strongly dissatisfied) to 5 (strongly satisfied).

*Household finances* was measured using two items. The *Costs of living in home* was determined by asking tenants “How easy or difficult is it for you to meet the costs of living in this home?” A 5-point answering scale ranged from “very easy” (1) to “very difficult” (5), with “neither easy nor difficult” as the scale midpoint (3). The *Difficulties paying utility bills* variable reflects responses to the question: “In the past 12 months, have you had difficulties paying scheduled utility bills, such as electricity, water, gas?” The response options were “yes” (1) and “no” (0).

Three mental, respiratory and general health outcomes were used in the study.

A *mental health* scale was created by averaging the answers to five questions relating to how much of the time in the previous 4 weeks respondents have been a very nervous person (reversed), have felt so down in the dumps that nothing could cheer them up (reversed), have felt calm and peaceful, have felt downhearted and blue (reversed), and have been a happy person. Scale analyses showed that the five items could be combined to create an internally consistent scale (Cronbach’s alpha ≥0.85 across the five surveys). The frequency response options ranged from “none of the time” to “all of the time”, which were subsequently weighted to form a scale ranging from 0 to 100.

*Respiratory health* was assessed by asking respondents to report whether they had experienced any of eleven listed respiratory symptoms in the past month. The symptoms included coughing, bringing up phlegm, shortness of breath, wheezing, chest tightness, runny nose, blocked nose, sinus swelling, sneezing, sore throat or fever. The responses were dichotomised, reflecting whether respondents had experienced *any respiratory symptoms* (1) or not (0).

The *general health* variable reflects responses to the question “In general, how would you say your health is?” Respondents could use the following categories: excellent, very good, good, fair, poor. This variable was dichotomised to contrast excellent, very good and good (1) with fair and poor (0) health. The self-rated health variable has been validated as a measure of general health and mortality [34–36].

### Statistical analysis

The five-wave repeated cross-sectional survey was analysed using multilevel modelling, with individual responses to the five surveys (level 1) nested within the properties that are part of the council’s housing stock (level 2). The nested multilevel design allowed for the time-dependent observations over the seven-year period of the study to be taken into account [37]. Analyses were conducted with the MLwiN 2.36 software package [38]. The multilevel approach allowed the simultaneous analysis of multiple properties at different stages of improvement during the upgrade programme, while dealing with repeated observations for individuals living in the intervention homes and unbalanced data with different numbers of observations for individuals in the properties. The final dataset used for the analyses consisted of 1980 properties with a single observation, 1476 properties with two observations, 968 properties with three observations, 442 properties with four observations, and 81 properties with five observations.

The analyses consisted of a series of multilevel models with the nine outcome measures as the dependent variables. The basic statistical model included the intervention measures as the independent variables, together with the year of the survey and the socio-demographic variables of gender, age, working and retirement status, possession of educational qualifications, reception of housing benefits, area deprivation (Welsh Index of Multiple Deprivation), and smoking status. The individual socio-demographics variables were included as dummy variables. The year of the survey was included as a number ranging from (year) 1 to (year) 8.

Separate series of models were constructed for each of the eight intervention measures, as well as for the *number of measures* and *total spend* variables. Parameters were estimated using restricted iterative generalised least squares (RIGLS) procedures. Different types of models were constructed dependent on the type of outcome variable. Linear regression models were used for the *satisfaction with state of repair*, *thermal comfort*, *costs of living in home*, and *mental health* outcome variables. Logistic regression models were used for the *suitability of housing*, *any building problems*, *difficulties paying bills*, any respiratory symptoms, and *good health* outcome variables.

In this paper we report the regression coefficients for the different intervention measures (including the number of measures and total spend variables). These coefficients show the extent to which each measure is individually associated with a change in the different outcome variables, taking account of the year of the survey and individual socio-demographic characteristics. The *number of measures* coefficient provides an estimate of the change in the outcome variable for each additional intervention measure installed, while the *total spend* coefficient provides an estimate of the change in the outcome variable for each £1000 invested under the upgrade programme. The latter two variables can therefore be used to link the extent of the intervention to the different social and health outcomes.

The associations of the covariates with the nine outcome variables are provided in Appendix. The ‘year’ coefficient in the table provides the underlying trend of the outcome variable over the 7 years of the study (in change/year);

## Results

### Descriptive results

Table 4 shows the nine outcome measures for the five surveys. Overall, there were no clearly discernible trends for the different measures over the seven-year period. While there were some differences between the 2009 survey on the one hand and all the other surveys on the other, these are likely to reflect differences in composition between the pilot study and the rest.

**Table 4:** Outcome measures for the five surveys (%)

Outcome measure		2009 (n = 2075)	2011 (n = 2219)	2012 (n = 2015)	2014 (n = 1991)	2016 (n = 1709)
Housing suitability, satisfaction, and quality						
Housing considered suitable		73	85	85	86	87
Satisfaction with state of repair	Very satisfied	25	36	34	42	48
	Fairly satisfied	37	33	36	32	26
	Neither/nor	12	10	9	7	7
	Dissatisfied	12	12	11	9	11
	Very dissatisfied	11	8	8	8	8
Any building problems		59	53	51	57	57
Thermal comfort and household finances						
Mean thermal satisfaction (SD)	Scale: 1–5	3.6 (1.3)	3.8 (1.3)	3.8 (1.2)	3.9 (1.3)	4.0 (1.3)
Meeting costs of living in home	Very easy	7	28	27	23	30
	Fairly easy	26	35	35	38	29
	Neither/nor	29	16	17	15	20
	Fairly difficult	23	12	12	16	15
	Very difficult	12	7	8	9	5
Difficulties paying bills		27	30	30	28	17
Mental, respiratory, and general health						
Mean mental health score (SD)	Scale: 0–100	62.2 (23.8)	66.5 (23.8)	67.2 (23.7)	67.3 (23.6)	66.6 (25.12)
Any respiratory symptoms		76	66	60	62	60
Good health		41	41	40	41	39

Note: the percentages in the table may not always add up to 100% due to rounding and missing values

### Housing suitability, satisfaction, and quality

Table 5 shows that the installation of most individual intervention measures were, as hypothesised, positively associated with perceptions of housing suitability, satisfaction, and quality. The installation of new kitchens, bathrooms, and electrics was also associated with a lower likelihood of reporting any housing problems, in contrast to what was hypothesised.

**Table 5:** Associations of the intervention measures with the housing suitability, satisfaction, and quality outcome measures

	Suitability of housing [B (SE)]	Satisfaction with state of repair [B (SE)]	Any building problems [B (SE)]
Windows and doors	0.143 (0.129)	0.096 (0.049)	−0.455 (0.097)***
Boilers	0.225 (0.086)**	0.201 (0.031)***	−0.101 (0.057)
Kitchens	0.486 (0.091)***	0.179 (0.042)***	−0.369 (0.060)***
Bathrooms	0.558 (0.091)***	0.368 (0.032)***	−0.331 (0.060)***
Electrics	0.396 (0.090)***	0.377 (0.032)***	−0.341 (0.060)***
Loft insulation	0.317 (0.086)***	0.122 (0.034)***	−0.053 (0.062)
Cavity wall insulation	0.025 (0.033)	−0.042 (0.035)	−0.009 (0.011)
External wall insulation	0.189 (0.111)	0.179 (0.042)***	−0.232 (0.075)**
Number of measures	0.132 (0.022)***	0.104 (0.008)***	−0.107 (0.015)***
Total spend	0.033 (0.006)***	0.028 (0.002)***	−0.031 (0.004)***

*p < .05, **p < .01, ***p < .001

A number of associations were not significant: the installation of new windows and doors was not associated with higher suitability and satisfaction ratings; external wall insulation was also not associated with higher suitability ratings; the installation of either boilers or loft insulation was not significantly associated with the reporting of any building problems; and external wall insulation was not significantly associated with any of the housing suitability, satisfaction, and quality outcome measures.

Table 5 further shows that the *number of measures* and *total spend* variables were both positively associated with the housing suitability, satisfaction, and quality outcome measures. A positive association was found between the number of measures variable and perceptions of housing suitability (OR = 1.14, 95% CI = 1.09–1.19) and satisfaction with the property’s state of repair (B = 0.104 (0.008), *p* < 0.001). The likelihood of respondents reporting any building problems reduced by 10% (OR = 0.90, 95% CI = 0.87–0.93) with each additional intervention measure installed.

Positive associations were found between the total spend variable and perceptions of housing suitability (OR = 1.03, 95% CI = 1.02–1.05) and satisfaction with the property’s state of repair (B = 0.028 (0.002), *p* < 0.001). For each £1000 invested, the likelihood of tenants reporting building problems reduced by 3% (OR = 0.97, 95% CI = 0.96–0.98).

### Thermal comfort and household finances

Table 6 shows that the installation of almost all individual intervention measures were associated with improvements in thermal comfort and household finances. In contrast to the expectations, this included the installation of new kitchens, bathrooms, and electrics.

**Table 6:** Associations of the intervention measures with the thermal comfort and household finances outcome measures

	Thermal comfort [B (SE)]	Costs of living in home [B (SE)]	Difficulties paying bills [B (SE)]
Windows and doors	0.161 (0.046)***	−0.293 (0.049)***	0.224 (0.097)*
Boilers	0.209 (0.029)***	−0.138 (0.030)***	−0.043 (0.063)
Kitchens	0.368 (0.032)***	−0.214 (0.031)***	−0.211 (0.065)**
Bathrooms	0.288 (0.030)***	−0.172 (0.031)***	−0.172 (0.065)**
Electrics	0.297 (0.030)***	−0.212 (0.031)***	−0.240 (0.065)***
Loft insulation	0.094 (0.032)**	−0.153 (0.032)***	−0.077 (0.066)
Cavity wall insulation	−0.015 (0.033)	0.025 (0.033)	0.007 (0.066)
External wall insulation	0.071 (0.039)	−0.103 (0.039)**	−0.115 (0.079)
Number of measures	0.090 (0.008)***	−0.072 (0.008)***	−0.044 (0.016)**
Total spend	0.024 (0.002)***	−0.017 (0.002)***	−0.012 (0.004)**

*p < .05, **p < .01, ***p < .001

A number of associations were non-significant: cavity wall insulation was not associated with any of the thermal comfort and household finances outcome measures; external wall insulation was not associated with thermal comfort; and the installation of new boilers was not associated with the *difficulties paying bills* outcome measure.

Table 6 further demonstrates that each additional intervention measure and £1000 invested were associated with improvements in thermal comfort (B = 0.090 (0.008), *p* < 0.001; and B = 0.024 (0.002), *p* < 0.001, respectively). The number of measures and total spend variables were also associated with better household finances. For each additional measure installed under the upgrade programme, tenants experienced fewer difficulties meeting the costs of living in their homes (B = −0.072; (0.008), *p* < 0.001) and were less likely to report difficulties paying scheduled utility bills (OR = 0.96, 95% CI = 0.93–0.99); and for each additional £1000 invested under the upgrade programme tenants experienced fewer difficulties meeting the costs of living in their homes (B = −0.017; (0.002), *p* < 0.001) and were less likely to report difficulties paying scheduled utility bills (OR = 0.99, 95% CI = 0.98–1.00).

### Mental, respiratory and general health

Table 7 shows that a majority of the intervention measures were associated with the health outcome measures. In most cases, these associations were positive, meaning that the interventions led to better mental health, fewer respiratory symptoms, and better general health. In contrast to the expectations, the installation of cavity wall insulation was associated with poorer mental (B = −2.052 (0.687), *p* < 0.05) and general (B OR = 0.78, 95% CI = 0.68–0.89) health, and an increase in reported respiratory symptoms (OR = 1.47, 95% CI = 1.30–1.66).

**Table 7:** Associations of the intervention measures with the health outcome measures

	Mental health [B (SE)]	Any respiratory Symptoms [B (SE)]	Good health [B (SE)]
Windows and doors	2.523 (0.950)**	−0.235 (0.101)**	−0.15 (0.099)
Boilers	1.975 (0.609)**	−0.008 (0.057)	0.122 (0.061)*
Kitchens	1.909 (0.632)**	−0.077 (0.060)	0.120 (0.063)
Bathrooms	1.660 (0.631)**	−0.102 (0.060)	0.089 (0.063)
Electrics	2.216 (0.638)***	−0.103 (0.060)	0.116 (0.064)
Loft insulation	4.281 (0.660)***	−0.145 (0.062)**	0.183 (0.066)**
Cavity wall insulation	−2.052 (0.687)*	0.385 (0.062)***	−0.253 (0.067)***
External wall insulation	2.657 (0.823)**	−0.416 (0.073)***	0.255 (0.079)**
Number of measures	0.826 (0.161)***	−0.023 (0.015)	0.035 (0.015)*
Total spend	0.223 (0.044)***	−0.011 (0.004)**	0.012 (0.004)**

*p < .05, **p < .01, ***p < .001

A number of associations were not significant: windows and doors were not associated with the likelihood of reporting good health, and boilers were not associated with the likelihood of reporting any respiratory symptoms.

In contrast to the expectations, new kitchens, bathrooms, and electrics were non-significantly associated with the likelihood of reporting good health.

Table 6 further shows that the *number of measures* and *total spend* variables were both associated with better mental, respiratory and general health. For each additional measure installed (B = 0.826 (0.161), *p* < 0.001) and £1000 invested (B = 0.223 (0.044), *p* < 0.001), tenants reported better mental health; for each £1000 invested OR = 0.99, 95% CI = 0.98–1.00) tenants were less likely to report any respiratory symptoms; and for each additional measure installed (OR = 1.03, 95% CI = 1.00–1.07) and £1000 invested (OR = 1.01, 95% CI = 1.00–1.02) tenants were more likely to report good health.

## Discussion

This paper examined changes in a range of social and health outcomes following upgrades to a national social housing standard, and whether these outcomes can be linked to specific intervention measures that were part of the upgrade programme. A majority of the individual intervention measures (i.e. new windows and doors; boilers; kitchens; bathrooms; electrics; loft insulation; and cavity/external wall insulation) were, as expected, positively associated with a number of social (i.e. housing suitability, satisfaction, and quality; thermal comfort and household finances) and health (mental, respiratory and general health) outcomes; and analyses revealed associations between the number of measures installed and the total amount invested on the one hand and the different social and health outcomes on the other. There were however a number of exceptions. Most notably, the installation of cavity wall insulation was associated with poorer health outcomes, and did not lead to higher suitability and satisfaction ratings, nor to improved thermal satisfaction and household finances. In contrast to what was expected, new kitchens, bathrooms and electrics were all associated with improved housing satisfaction, thermal comfort and household finances. Kitchens and bathrooms had positive associations with mental health, they were not linked to better general health. Also, no association was found between the number of measures installed and respiratory health.

The results suggest that managed programmes to meet national housing standards may result in a range of positive social and health outcomes, and that these improvements are generally proportionate to the number of measures installed and amount invested. There may however be risks associated with specific measures. The results relating to cavity wall insulation appear in line with other recent research, suggesting that energy efficiency measures may pose risks to respiratory health in certain circumstances [22]. It is significant that, while cavity wall insulation–which was installed without any additional ventilation– was linked to poorer health outcomes in this study, external wall insulation–which was accompanied by extractor fans– was associated with better health outcomes. This, together with a recent monitoring study that found that cavity wall insulation (without extractor fans) increased indoor humidity levels but external wall (with extractor fans) did not [12], indicates that ventilation as part of energy efficiency upgrades requires more attention to avoid any negative outcomes resulting from reduced ventilation and air exchange rates. It is important to note that the research involved repeated cross-sectional surveys, and that more research is needed to firmly establish causality.

Other results that require further attention relate to the unexpected positive links between new kitchens, bathroom and electrics with the reduced reporting of housing-related problems, such as damp and mould, and improved reports of thermal comfort and household finances. While, theoretically, there are no clear reasons to believe that these measures can eliminate damp and mould and/or substantially improve thermal comfort and household finances, the effects found in this study were sizeable. This may be linked to mechanical ventilation that was installed as part of the electrical upgrade element.

The research resonates well with other recent research. Grey and colleagues found that energy-efficiency investments increased subjective wellbeing and were linked to a number of psychosocial intermediaries that are conducive to better health; although they did not find changes in respiratory or physical health. [11] It is also in line those that observed improvements in thermal comfort and reductions in financial stress through quantitative [12–14] and qualitative [10, 13, 15, 17] work. There is however only limited work on outcomes following improvements other than affordable warmth interventions. There are clear parallels with work conducted as part of the GoWell programme in Glasgow, not only in terms of the content of the upgrade programme but also in terms of the results [4, 8]. The current paper, just as Curl and Kearns [4] followed a long-term housing improvement programme, and tried to disentangle the effect of different housing improvement works. However, rather than re-arranging multiple waves of data into two time periods representing a before and an after measurement, this current study took a multilevel modelling approach to make use of the complete dataset. This approach to analyse the repeated cross-sectional survey allows the different outcomes of the individual measures and the extent of the intervention to be compared with outcomes for tenants of houses that did not receive an intervention, while taking account of the time-dependent nature of the observations. It may therefore be useful for organisations that want to evaluate long-term upgrade programmes that do not lend themselves for randomisation or a stepped wedge design [39]. In this case, the council agreed through tenant consultation that the programme was to be spread evenly across the authority and with as little disruption to the occupants as possible. This shows that evaluations of complex non-health interventions cannot be separated from the practicalities of delivering the actual programme and from other social and economic considerations, and that different methodological and analytical approaches are needed that take account of a range of dimensions of complexity [40].

While the approach presented in this paper provides a statistical way in which a complex, long-term intervention can be evaluated, it is not without limitations. Even if the intervention was distributed across the council’s housing stock as evenly as possible, and delivered independent of need and initial housing condition, the intervention was not randomised. As discussed above, the study design and analytical approach was chosen to effectively capture the rolling programme of multiple housing intervention elements that were delivered over the seven-year period of the research. Furthermore, the improvements were delivered through four broad work packages. While the properties did not always receive all elements of a work package, multiple elements were often installed at the same time. This makes it difficult to disentangle the effects of the different individual measures. The survey frequency (surveys were conducted every one or 2 years) makes it difficult to attribute the changes to specific measures. Multiple work packages could have been delivered between the different waves of data collection. This means that some of the effects reported in this paper may reflect the effects of multiple elements that were delivered together.

The research was conducted over an extended seven-year period with a changing population. A high churn rate typical for social housing tenants complicates capture of the effects of the intervention at the individual level. The multilevel approach does however allow adjustments for changes in differences in socio-demographic composition across the subsequent waves of data collection. Just as with any study using survey methodology, sample bias could be an issue; although response rates were generally higher than in other studies that were conducted in low-income areas [11, 41]. Response bias could be resolved through data linkage, which was done in a related study. [7] While we did not assess the condition of the houses at the start of the study, we did consider the extent of the intervention. That is, we examined the extent to which the number of measures installed and amount invested were linked to the different social and health outcomes. It is possible to use these results as an indicator of the effects that can be expected from the size of the investments to upgrade housing stock. The population studied is typical for those living in social housing in the UK; and similar housing standards have been rolled out across the different UK countries. The study focused specifically on standard UK social housing stock, as responses from non-traditional housing were excluded from the analyses. We therefore expect that the results of the study is generalisable to social housing residents in temperate regions living in homes that do not meet accepted housing quality standards.

## Conclusion

The multilevel analysis of repeated cross-sectional surveys, which followed a managed upgrade programme designed to meet a national housing standard, found evidence that area-based investment in housing can provide a number of measurable benefits to tenants. Not only were several measures associated with better mental, respiratory and general health outcomes, they also resulted in fewer building-related problems, improved thermal comfort and household finances, and higher residential satisfaction. While the gains associated with each additional measure were generally modest, gains could add up with sizeable investments. There may however be some health risks associated with specific measures. In particular, the study suggests that mechanical ventilation requires more attention when upgrading the energy efficiency through fabric work. Overall, though, the study shows that the social and health outcomes of managed housing upgrade programme are predominantly positive, and that that they are proportionate to the number of measures installed and amount invested.

## Appendix

**Table 8:** Associations of the covariates with the outcome mesures

	Housing satisfaction	Housing satisfaction	Housing quality	Thermal comfort	Household finances		Mental health	Respiratory health	General health
	Suitability of housing [B (SE)]	Satisfaction with state of repair [B (SE)]	Any. building problems [B (SE)]	Thermal comfort [B (SE)]	Costs of living in home [B (SE)]	Difficulties paying bills [B (SE)]	Mental health [B (SE)]	Any respiratory symptoms [B (SE)]	Good health [B (SE)]
Male	−0.013 (0.084)	−0.046 (0.030)	−0.127 (0.054)*	0.037 (0.028)	−0.043 (0.029)	−0.137 (0.061)*	2.410 (0.576)***	−0.039 (0.055)	0.059 (0.058)
Age 36–45	0.196 (0.116)	−0.060 (0.053)	−0.001 (0.105)	0.130 (0.049)**	0.101 (0.051)*	−0.027 (0.096)	−7.338 (1.002)***	0.191 (0.098)*	−0.835 (0.106)***
Age 46–54	0.529 (0.125)***	−0.209 (0.053)***	−0.397 (0.101)***	0.249 (0.050)***	0.037 (0.051)	--0.216 (0.097)*	−6.403 (1.013)***	0.313 (0.098)	−1.554 (0.108)***
Age 55–64	0.734 (0.130)***	−0.375 (0.052)***	−0.507 (0.098)***	0.405 (0.049)***	−0.153 (0.050)**	−0.570 (0.099)***	−3.346 (1.007)***	0.503 (0.098)**	−1.801 (0.107)***
Age 65+	1.073 (0.168)***	−0.628 (0.060)***	−0.992 (0.112)***	0.601 (0.051)***	−0.443 (0.059)***	−1.115 (0.121)***	1.659 (1.180)	0.451 (0.113)***	−2.049 (0.125)***
Not working	0.358 (0.108)***	−0.005 (0.043)	−0.087 (0.082)	0.035 (0.040)	0.028 (0.032)	0.258 (0.084)**	−12.051 (0.823)***	0.192 (0.080)*	−1.547 (0.088)***
Retired	0.344 (0.170)*	−0.197 (0.057)***	−0.366 (0.105)***	0.154 (0.053)**	−0.122 (0.056)*	−0.048 (0.119)	−3.175 (1.113)**	−0.077 (0.107)	−0.630 (0.114)***
No qualification	0.289 (0.084)***	−0.219 (0.031)***	−0.309 (0.057***)	0.182 (0.029)***	−0.194 (0.030)***	−0.122 (0.062)	0.980 (0.593)	−0.388 (0.098)	−0.213 (0.059)***
Housing benefits	−0.108 (0.096)	−0.009 (0.033)	−0.039 (0.062)	0.097 (0.031)**	0.028 (0.032)	0.536 (0.072)***	−3.739 (0.650)***	0.092 (0.062)	−0.230 (0.065)***
WIMD	0.210 (0.042)***	−0.031 (0.016)	−0.006 (0.028)	0.056 (0.015)	0.018 (0.015)	−0.099 (0.031)**	1.050 (0.311)***	−0.090 (0.028)**	0.045 (0.030)
Current smoker	−0.184 (0.092)*	0.251 (0.034)***	0.339 (0.062)***	−0.238 (0.032)***	0.392 (0.033)***	0.473 (0.031)***	−3.515 (0.648)***	0.463 (0.062***)	−0.232 (0.067)***
Past smoker	−0.295 (0.100)**	0.236 (0.034)***	0.480 (0.063)***	−0.179 (0.034)***	0.377 (0.033)***	0.190 (0.073)**	−0.403 (0.666)	0.563 (0.064)***	−0.289 (0.067)***
Year	0.050 (0.016)***	−0.058 (0.006)***	−0.009 (0.011)	0.079 (0.005)***	−0.076 (0.006)***	−0.085 (0.012)***	0.657 (0.109)***	−0.127 (0.011)***	−0.002 (0.011)

*p < .05, **p < .01, ***p < .001

## Footnotes

1 The Standard Assessment Procedure (SAP), developed by the Building Research Establishment, is the UK Government’s recommended method system for measuring the energy rating of residential dwellings [42]. The Welsh Housing Quality Standard requires a minimum rating of 65 out of 100.

2 LSOAs is part of a geographical hierarchy used to report official small-area statistics in Wales and England.

3 The *windows and doors* were double glazed and had safety features complying with *Secured By Design* (http://www.securedbydesign.com).

4 Both *kitchens and bathrooms* need to be up-to-date with appropriate design, layout and state of repair for their safe use.

5 The *electrics* element involved rewiring (where necessary), the installation of power sockets, Carbon Monoxide and Smoke detectors, security lighting, and extractor fans in kitchens and bathrooms.

6 The *insulation* measures were selected to achieve a minimum SAP (Standard Assessment Procedure for Energy Rating of Dwellings 2005) rating of 65 out of 100. External wall or cavity wall insulation were installed depending on the building type. External wall insulation was companied by mechanical ventilation, while cavity wall insulation was not.

7 Data were collected in the following periods: Survey 1: 23 February to 20 March 2009; Survey 2: 14 February to 2 April 2011; Survey 3: 13 February to 31 March 2012; Survey 4: 1 February to 23 March 2014 and Survey 5: 15 February to 5 April 2016.

8 Response rates for the five surveys were as follows (excluding non-traditional properties):

**Table:**

Survey	Year	Type	Contacted	Achieved sample	Response rate
1	2009	Face-to-face	553	319	55%
		Postal	8237	1769	21%
2	2011	Face-to-face	2088	976	47%
		Postal	6180	1246	20%
3	2012	Face-to-face	2117	1034	49%
		Postal	6017	1008	17%
4	2014	Face-to-face	1989	1021	58%
		Postal	6039	972	16%
5	2016	Face-to-face	1769	906	51%
		Postal	5920	807	14%

9 New windows and doors: £3500; boilers: £5752; kitchens: £3298; bathrooms: £2349; electrics (re-wiring): £3159; loft insulation: £225; cavity-wall insulation: £1000; and external wall insulation: £9000.
